# The Relationship between Physical Exercise and Smartphone Addiction among Chinese College Students: The Mediating Effect of Core Self-Evaluation

**DOI:** 10.3390/bs13080647

**Published:** 2023-08-03

**Authors:** Yanbin Gong, Haibo Yang, Xuejun Bai, Yuehua Wang, Jiayu An

**Affiliations:** 1 College of Humanities and Law, Taiyuan University of Technology, Taiyuan 030024, China; 2 Faculty of Psychology, Tianjin Normal University, Tianjin 300387, China; yanghaibo@tjnu.edu.cn; 3 Tianjin Social Science Laboratory of Students’ Mental Development and Learning, Tianjin 300387, China; 4 Key Research Base of Humanities and Social Sciences of the Ministry of Education, Academy of Psychology and Behavior, Tianjin Normal University, Tianjin 300387, China; 5 Student Mental Health Guidance Center, Taiyuan University of Technology, Taiyuan 030024, China; wangyuehua@tyut.edu.cn; 6 College of Foreign Languages, Taiyuan University of Technology, Taiyuan 030024, China; anjiayu1276@tyut.edu.cn

**Keywords:** smartphone addiction, physical exercise, core self-evaluation, college students

## Abstract

Smartphone addiction is widespread among college students. Physical exercise and core self-evaluation are two potential factors that may influence smartphone addiction. This study aimed to investigate the relationship between physical exercise and college students’ smartphone addiction, as well as the mediating effect of core self-evaluation. Here, 643 undergraduate university students are surveyed via questionnaire using the Physical Activity Rating Scale, the Smartphone Addiction Scale for College Students, and the Core Self-Evaluations Scale. The participants include 363 males (56.5%) and 280 females (43.5%), with ages ranging from 17 to 25 years old (mean = 19.68, SD = 1.40). The obtained data are analyzed using SPSS26.0 and the PROCESS plugins. The main findings of the study are as follows: (1) There is a significant negative correlation between physical exercise and smartphone addiction (r = −0.30, *p* < 0.01), a significant positive correlation between physical exercise and core self-evaluation (r = 0.25, *p* < 0.01), and a significant negative correlation between core self-evaluation and smartphone addiction (r = −0.52, *p* < 0.01). (2) There is a mediating effect of core self-evaluation between physical exercise and smartphone addiction. The current study can provide new evidence for the impact of physical exercise on smartphone addiction and highlights the importance of core self-evaluation. Moreover, research ideas and methodological guidance are provided for the following interventions and treatments targeting college students’ smartphone addiction.

## 1. Introduction

Smartphone use is like a “double-edged sword”, which has brought great convenience to people’s study, work, and life, but also contributes to the problem of smartphone addiction, which refers to the user’s psychological or behavioral problems caused by smartphone abuse [1]. Previous studies have shown that mobile phone addiction has negative effects on academic performance [2,3,4], sleep quality [5,6], and interpersonal adaptation [7,8]. Moreover, there is a significant positive correlation with loneliness [9,10], depression [11,12], anxiety [13,14], and other negative emotions. According to the 51st Statistical Report on China’s Internet Development released by the China Internet Network Information Center, by December 2022, the number of mobile internet users in China had reached 1.067 billion, with the average person surfing 26.7 hours per week. At present, smartphone addiction is widespread among college students [15,16]. It is found that college students’ mobile phone addiction is influenced by many factors, such as family functions [17], social support [18], trait mindfulness [19], and values [20].

Moreover, the World Health Organization officially declared that the COVID-19 pandemic was no longer a “public health emergency of international concern” on 5 May 2023, but the continuous spread of COVID-19 for more than three years not only poses a threat to people’s lives and physical health, but also has a significant impact on people’s mental health. It even changes the way people study, work, and live. College students took online courses under the background of an initiative entitled “ensuring learning to continue when classes are disrupted”, and reported information related to the pandemic through WeChat groups and QQ groups, all of which have increased the frequency of mobile phone use to some extent. In addition, most college students have a great demand for social contact and entertainment. Under the circumstances, mobile phones that can connect to the internet have become the best “device”, and the time spent by college students on mobile phones continues to rise. The prevention and intervention of college students’ smartphone addiction have become a practical problem and an important topic to be addressed urgently.

### 1.1. Physical Exercise and Smartphone Addiction

Physical exercise, by means of exercise load, includes fitness, recreation, rehabilitation care, and mental intelligence exercise, aiming at enhancing physical fitness, improving physical and mental health, and maintaining physical ability [21]. Physical exercise is an important means of promoting individual mental health [22,23], as confirmed by Nie et al. [22], who examined the relationship between physical exercise and mental health during the COVID-19 outbreak in China, and found that physical exercise could significantly improve mental health in all age groups. However, there is insufficient evidence to support whether physical exercise can reduce smartphone addiction among Chinese college students.

According to self-determination theory [24], the basic drive of human beings is to seek the satisfaction of psychological needs. Human beings have three basic psychological needs: autonomy (a need to determine his or her own behavior without being coerced or interfered with by others), relatedness (a need to establish and keep close relationships with others), and competence (a need to have the ability to finish important matters). Recently, with the development of smartphone technology, various smartphone apps play an increasingly important role in satisfying the basic psychological needs of individuals; for example, WeChat and TikTok help to satisfy the individual’s need for autonomy and relatedness, and mobile games help to satisfy the individual’s need for autonomy, competence, and relatedness. All three basic psychological needs of an individual can be met to varying degrees by the use of a smartphone. If college students fail to fulfill their basic psychological needs in the real world, they may turn to their smartphones for compensation, leading to smartphone addiction. In the real world, college students’ active participation in physical activity not only helps to reduce the time spent on smartphones, but also satisfies the three basic psychological needs of individuals. Specifically, college students are able to freely choose the content and types of physical exercise (autonomy need), master one or more motor skills by themselves (competence need), and facilitate interpersonal interactions (relatedness need), in turn reducing the probability of the occurrence of smartphone addiction.

In recent years, the impact of physical exercise on smartphone addiction has attracted widespread attention. An increasing amount of empirical research has focused on the relationship between physical exercise and smartphone addiction. Previous studies have shown that mobile phone addiction can be improved through physical exercise; for example, Wang et al. recruited 756 students from two middle schools aged between 12 and 18 years, and found that physical activity can negatively predict mobile phone dependence [25]. Moreover, some studies have shown that physical exercise can improve the mobile phone addiction of Chinese college students [26,27,28,29]; for example, Zhong et al. also found that physical activity can negatively predict mobile phone dependence [26], and Li et al. found that physical exercise can negatively predict the mobile phone addiction of college students [27]. However, the measurement tools for mobile phone addiction used in these studies did not distinguish between non-smartphones and smartphones [26,27,28,29]. Therefore, there is insufficient research evidence to support whether physical exercise can reduce smartphone addiction among Chinese college students. Smartphone addiction and internet addiction are both non-drug addictions or behavioral addictions. Since the smartphone itself is a new type of internet medium, and individuals use their smartphones for activities such as information gathering, online communication, and online games, there may be similar addiction mechanisms between smartphone addiction and internet addiction. Previous studies have shown that physical exercise is effective in reducing internet addiction problems [30], so it is reasonable to hypothesize that physical exercise is also effective in reducing smartphone addiction problems. In addition, relevant studies have shown that physical exercise can improve the problem of mobile phone addiction among college students [26,27,28,29]. Based on the above research results, this study puts forward the first hypothesis:

**Hypothesis** **1.** *Physical exercise has a significant negative effect on college students’ smartphone addiction.*

### 1.2. Mediating Effects of Core Self-Evaluation

Core self-evaluation refers to the most basic evaluation made by individuals on their own abilities and values, which is put forward by Judge et al. [31]. It is a good predictor of career adaptability [32], job satisfaction [33], and work engagement [34].

In order to better prevent and intervene in the smartphone addiction of college students, it is insufficient to only explore the direct relationship between physical exercise and smartphone addiction—it is also necessary to examine the internal mechanism between the two. Referring to some previous studies, this study examines the mediating effects of core self-evaluation on the relationship between physical exercise and smartphone addiction among college students.

At the theoretical level, the cognitive-behavioral model of pathological internet use proposed by Davis puts forward that individual inappropriate self-cognition, such as self-doubt and negative self-evaluation, are risk factors leading to the emergence and development of internet addiction [35]. Smartphone addiction and internet addiction are both non-drug addictions or behavioral addictions. Since the smartphone itself is a new type of internet medium, and individuals use their smartphones for activities such as information gathering, online communication, and online games, there may be similar addiction mechanisms between smartphone addiction and internet addiction. Based on the cognitive-behavioral model, we suppose that, on the one hand, core self-evaluation, as a cognitive factor, may have a negative predictive effect on smartphone addiction in college students, and the lower an individual’s core self-evaluation is, the more likely he or she is to be addicted to smartphone; on the other hand, physical exercise, as a normalized and non-threatening life event, may have an impact on smartphone addiction through an individual’s cognitive processes (e.g., core self-evaluation). To sum up, it can be reasonably speculated that physical exercise may indirectly affect smartphone addiction, where core self-evaluation may be the mediating variable. A recent empirical study shows that physical exercise has a positive predictive effect on core self-evaluation [36]. Furthermore, core self-evaluation has a negative predictive effect on mobile phone addiction [37,38,39,40]. In addition, it is shown that core self-evaluation plays mediating effects in many relationships, through which loneliness has an impact on college students’ mobile phone addiction [41], and technological interference has an impact on teenagers’ smartphone addiction [42]. Therefore, this study puts forward the following hypothesis:

**Hypothesis** **2.** *There is a mediating effect of core self-evaluation between physical exercise and smartphone addiction.*

**Figure 1:**
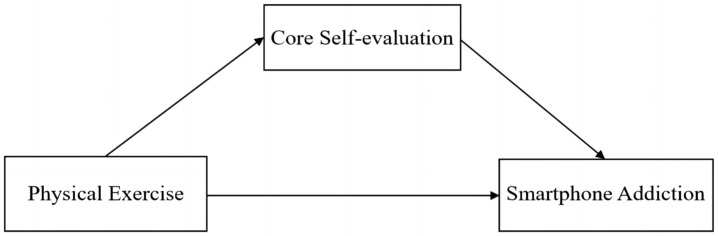
Hypothesis model of the mediating effect of core self-evaluation in the relationship between physical exercise and smartphone addiction among college students.

Although it is speculated that there is a close relationship between core self-evaluation, physical exercise, and smartphone addiction among college students based on the analysis of the existing literature, there is insufficient direct evidence on the mechanism of core self-evaluation between physical exercise and smartphone addiction. Therefore, in view of the existing empirical research results, self-determination theory, and the cognitive-behavioral model of pathological internet use, this study intends to establish a mediating effect model, which is shown in Figure 1, to explore the impact of physical exercise on smartphone addiction, and to investigate the mediating effect of core self-evaluation, which is hoped to provide an empirical basis for the scientific prevention and effective intervention of smartphone addiction for college students. On the one hand, the stability of the effect of physical exercise on college students’ smartphone addiction can be verified, and its potential mechanism can be further investigated. On the other hand, effective strategies to improve college students’ smartphone addiction and core self-evaluation can be explored.

## 2. Method

### 2.1. Participants

A total of 827 questionnaires were collected through the Wenjuanxing survey platform among undergraduate students from a university in central China. In order to keep the questionnaire valid, the inclusion criteria of the valid questionnaire were defined as follows: (1) the questionnaire is complete or the information is not obviously wrong (for example, the age is 88 years old); (2) the IP addresses of the respondents are confined to the location of the school, and each IP address can only be used once; and (3) the time taken to fill in the questionnaire is between 1.5 and 15 min. A total of 643 valid questionnaires were collected, with a 77.75% response efficiency, including 363 males (56.5%) and 280 females (43.5%). The age range of the study subjects was 17–25 years, with a mean age of 19.68 years (SD = 1.40). This study was approved by the Research Ethics Committee of the College of Humanities and Law, Taiyuan University of Technology, China. We informed the participants that personal information obtained from the questionnaires would be kept strictly confidential and all results would be used for the study. Informed consent was obtained prior to the completion of the questionnaires.

### 2.2. Measures

In this study, we adopted mature scales to measure the variables.

#### 2.2.1. Smartphone Addiction Scale for College Students

Smartphone addiction was measured using the Smartphone Addiction Scale for College Students compiled by Su et al. [1]. Previous studies have proven that this scale is applicable to samples of Chinese college students, and has good reliability and validity [43,44]. The scale consists of 22 items, including six factors. There are seven items for withdrawal behavior, three items for salience behavior, three items for social comfort, four items for negative effects, three items for use of apps, and two items for renewal of apps. The items in the scale include “I always pay attention to updates for the applications already on my smartphone and keep them up-to-date” and “when my smart phone cannot connect to the Internet or receive a signal, I become anxious and my temper becomes irritable”. The answers of the scale were ranked by following the principle of a five-point Likert scale (“strongly agree” = 5 points). The higher the total score of the 22 items, the higher the level of smartphone addiction. In this study, the Alpha coefficient for this scale was 0.92.

#### 2.2.2. Physical Activity Rating Scale (PARS-3)

Physical exercise was measured using the Chinese version of the Physical Activity Rating Scale [45]. Previous studies have proven that this scale is applicable to samples of Chinese college students, and has good reliability and validity [27,28,29]. The scale has three items measuring exercise intensity, exercise frequency, and single exercise time, respectively. For example, “what is the intensity of your physical exercise” and “how frequently do you engage in the above physical activities per month” are listed in the scale. According to Liang’s recommendations, physical exercise score = exercise intensity score × (exercise time score − 1) × exercise frequency score. After the measurement, the Alpha coefficient for PARS-3 in this study was 0.69.

#### 2.2.3. Core Self-Evaluation Scale

Core self-evaluation was measured using the Chinese version of the Core Self-Evaluation Scale revised by Du Jianzheng et al. [46]. Previous studies have demonstrated that this scale is applicable to samples of Chinese college students [38,39,40]. The Chinese version of the Core Self-Evaluation Scale has been shown to be valid and efficient for measuring core self-evaluation. The scale has a single factor structure composed of 10 items, including “I believe that I can achieve success in life” and “I feel that many things are going badly and there is no hope”. Each item is rated on a five-point Likert scale (“strongly agree” = 5 points). The higher the total score of all questions, the higher the core self-evaluation of the individual. In this study, the Alpha coefficient for this scale was 0.89.

### 2.3. Statistical Analysis

In this study, the questionnaire tests were organized in classes by trained investigators. Under the regulated guidance, the participants were informed of the requirements and precautions. All personal information obtained during the questionnaire tests was kept strictly confidential. After finishing the questionnaire tests, the completeness was checked. The acquired data were statistically analyzed using IBM SPSS Statistics 26.0 and the PROCESS plug-in [47]. The common method bias was examined using Harman’s one-factor test, the relationship between the main variables was investigated using Pearson correlation analysis, and the difference in the main variables caused by gender was tested using an independent sample *t*-test. In addition, Model 4 in the PROCESS macro was used to analyze the mediating effects of the core self-evaluation. The 95% confidence intervals for the mediating effects were calculated by Bootstrap repeated sampling 10,000 times, and *p* < 0.05 was considered statistically significant.

## 3. Results

### 3.1. Common Method Bias Test

There is an appropriate amount of reverse scoring questions to reduce the influence of common method biases. Before releasing the questionnaires, the participants were informed to fill in the questionnaires anonymously and that the results were only used for academic studies. However, the results related to physical exercise, smartphone addiction, and core self-evaluation were derived from self-reports, so there may be common method bias. Therefore, Harman’s one-factor test was conducted to test common method bias after collecting the data. Exploratory factor analysis was carried out on all items of the Smartphone Addiction Scale for College Students, the Physical Activity Rating Scale, and the Core Self-Evaluation Scale. The results showed that there were seven factors with characteristic roots greater than 1, and the variance interpretation rate of the first common factor was 31.10%, which was less than the critical value of 40%, indicating that there was no significant common method bias in this study [48,49], and subsequent data analysis could be conducted.

### 3.2. Gender Differences of Physical Exercise, Smartphone Addiction, and Core Self-Evaluation

The role of gender was one of the main concerns in this study. The results of the gender differences in physical exercise, smartphone addiction, and core self-evaluation can be seen in Table 1. The independent sample *t*-test was further used to show the gender differences in physical exercise, smartphone addiction, and core self-evaluation. In the independent sample *t*-test, gender was the independent variable and smartphone addiction, physical exercise, and core self-evaluation were the dependent variables. The results showed that there were significant gender differences in terms of physical exercise, and the physical exercise level of male students was significantly higher than that of female students (t = 8.53, *p* < 0.001). There were significant gender differences in smartphone addiction, and the level of smartphone addiction in females was significantly higher than that in males (t = 1.97, *p* < 0.05). There was no significant gender difference in terms of core self-evaluation.

Gender differences have been a concern in psychological research. This study found significant gender differences in physical exercise and smartphone addiction, and insignificant gender differences in core self-evaluation. However, it is unclear whether gender has a moderating effect on the relationships among physical activity, core self-evaluation, and smartphone addiction. Therefore, this study will further explore the moderating role of gender in subsequent analyses.

### 3.3. Descriptive Statistics and Correlation Analysis of Each Variable

The results of Pearson’s correlation analysis for physical exercise, smartphone addiction, and core self-evaluation can be seen in Table 2. It is found that physical exercise was significantly negatively correlated with smartphone addiction (r = −0.30, *p* < 0.01), physical exercise had a positive correlation with core self-evaluation (r = 0.25, *p* < 0.01), and there was a significant negative correlation between core self-evaluation and smartphone addiction (r = −0.52, *p* < 0.01), which indicated that the relationship among the three variables was close and can be further analyzed on the role of mediation.

### 3.4. Mediating Effect of Core Self-Evaluation

To further investigate the internal mechanism of physical exercise on smartphone addiction among college students, we first examined the mediating effect of core self-evaluation on the relationship between physical exercise and smartphone addiction among college students. We constructed the mediating effect model of core self-evaluation between physical exercise and smartphone addiction, taking physical exercise as the independent variable, core self-evaluation as the mediator variable, age and gender as the control variable, and smartphone addiction as the dependent variable. All continuous variables in this study were standardized. In the study, Model 4 in the PROCESS macro program developed by Hayes was used to analyze the mediation effect of core self-evaluation, to repeat the sampling 10,000 times, and to calculate the 95% confidence interval of the mediation effect. If 0 is not included in the interval, it means that the mediating effect of the mediating variable is significant; otherwise, it means that the effect is not significant [50].

**Figure 2:**
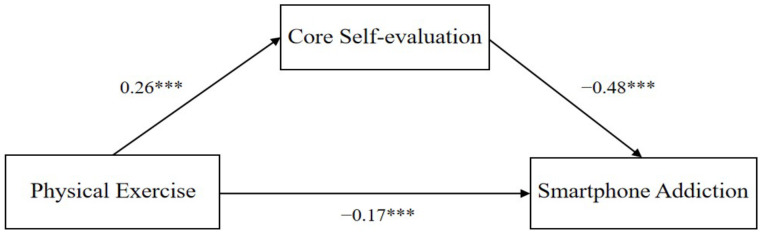
Mediating model of core self-evaluation in the relationship between physical exercise and smartphone addiction among college students. (*** *p* < 0.001).

The regression analysis results in Figure 2 (*** *p* < 0.001) reveal that physical exercise has a significant positive prediction effect on core self-evaluation (β = 0.26, t = 6.32, *p* < 0.001) and core self-evaluation has significant negative prediction effect on smartphone addiction (β = −0.48, t = −13.90, *p* < 0.001). Physical exercise is shown to have a significant negative prediction effect on smartphone addiction among college students after adding core self-evaluation as a mediator variable (β = −0.17, t = −4.52, *p* < 0.001).

Table 3 shows the analysis results of the mediating effect of core self-evaluation on the relationship between physical exercise and smartphone addiction. As is shown in this table, the 95% confidence interval of the direct effect does not contain 0 (95%CI = [−0.24, −0.09]), which indicates that there is a significant direct effect; that is, physical exercise can directly affect the smartphone addiction of college students, and the effect value of the direct path from physical exercise to smartphone addiction is −0.17. Moreover, the 95% confidence interval of the indirect effect does not contain 0 (95%CI = [−0.17, −0.07]), suggesting that there is a significant indirect effect between physical exercise and smartphone addiction, with an effect value of −0.12 for the indirect path from physical exercise to core self-evaluation to smartphone addiction.

It is found that core self-evaluation has a partial mediating effect between physical exercise and smartphone addiction among college students, and its mediating effect accounts for 41.38% of the total effect. Based on the above findings, the mediating role model of core self-evaluation hypothesized in this study between physical exercise and college students’ smartphone addiction has been verified.

Moreover, psychological research has paid great attention to gender differences, but the moderating effect of gender on the relationships among physical exercise, core self-evaluation, and smartphone addiction should be further discussed. Therefore, we should further test the moderating effect of gender on the relationships among physical exercise, core self-evaluation, and smartphone addiction. In the moderating effect analysis, physical exercise is the independent variable, the smartphone addiction of college students is the dependent variable, core self-evaluation is the mediator variable, age is the control variable, and gender is the moderator variable. Model 59 in the PROCESS macro developed by Hayes was used for examining the moderating role of gender. Model 59 assumed that all paths of the mediation model are moderated. The results found that the moderating effects of gender on the paths of mediation model are insignificant with the 95% confidence interval containing 0 and *p* > 0.05. The results meant that there were no differences between gender groups in the relationships among physical exercise, core self-evaluation, and smartphone addiction among college students.

## 4. Discussion

In this study, three scales were used to explore the effects of physical exercise on smartphone addiction, and also the mediating effect of core self-evaluation, in order to provide some reference and basis for the prevention of and intervention in smartphone addiction and for future research.

### 4.1. Physical Exercise and Smartphone Addiction in College Students

Many studies, such as Li et al. [27], found that physical exercise can negatively predict mobile phone addiction in college students, which indicates that mobile phone addiction among college students can be improved through physical exercise [27,28,29]. However, the measurement tools for mobile phone addiction used in these studies did not distinguish between non-smartphones and smartphones [26,27,28,29]. This study employed scales [1] that differed from those that did not distinguish between non-smartphones and smartphones, adding two dimensions of APP use and APP update. The findings indicate that physical exercise has a significant negative predictive effect on college students’ smartphone addiction, confirming study hypothesis 1 and indicating that physical exercise helps to improve the problem of smartphone addiction among college students, which supports the existing conclusions [27,28,29]. The results of this study also further support self-determination theory. The basic psychological needs of college students that are not satisfied in real life are risk factors for smartphone addiction. Smartphone addiction is most likely to occur when an individual’s three basic psychological needs are less satisfied in the real world and more satisfied in the virtual world. It has been shown that physical exercise has a significant positive predictive effect on basic psychological need satisfaction in college students [51]. Physical exercise reduces the possibility of smartphone addiction in college students by enhancing the satisfaction of their basic psychological needs. Moreover, both smartphone addiction and internet addiction are non-drug or behavioral addictions [52], and exercise can improve the human body’s dopamine signaling ability, affecting individuals’ addiction status [53], which can also explain the relationship between physical exercise and smartphone addiction among college students.

### 4.2. Mediating Effect of Core Self-Evaluation

There is a lack of direct evidence to show the mechanism of core self-evaluation between physical exercise and college students’ smartphone addiction. This study found that physical exercise has an impact on college students’ smartphone addiction via the mediating effect of core self-evaluation, specifically as follows: Physical exercise positively predicts the level of core self-evaluation, and the higher level of core self-evaluation represents the lower likelihood of smartphone addiction among college students. With a 41.38% effect size, the above findings validate study hypothesis 2 that core self-evaluation plays a partly mediating function between physical exercise and smartphone addiction among college students.

On the one hand, this study found that physical exercise can significantly positively predict core self-evaluation, and the results further support the previous research [36]. Physical exercise can increase an individual’s core self-evaluation level for the following reasons. Physical exercise can enhance individual resilience [54,55], which is a significant positive predictor of core self-evaluation [56]. Moreover, external stimuli can lead to obvious or subtle, temporary or permanent changes in core self-evaluation [57].

On the other hand, this study discovered that core self-evaluation can negatively predict smartphone addiction significantly, which further validates the findings of previous studies [37,38,39,40]. Core self-evaluation can positively predict the sense of life significantly [58], which can significantly improve the problem of mobile phone addiction in college students [59,60], so core self-evaluation is beneficial for improving college students’ smartphone addiction. In addition, the findings of this study may be explained by Davis’s cognitive-behavioral model of pathological internet use [35]. The model proposes that individual inappropriate self-cognition, such as self-doubt and negative self-evaluation, are risk factors for the emergence and development of internet addiction. Both smartphone addiction and internet addiction are non-drug or behavioral addictions [52]. As a cognitive factor, core self-evaluation can influence the formation of smartphone addiction and individuals with lower core self-evaluation scores are more likely to develop smartphone addiction.

### 4.3. Gender Differences

The main results of this study are as follows. (1) The physical exercise level of male students was significantly higher than that of female students, which is consistent with the results of previous studies [61]. Although the COVID-19 pandemic in the past three years has seriously affected the study and life of college students, male students take more advantage of physical exercise to relieve pressure than female students, and they invest more time and energy in sports activities [62]. (2) The level of smartphone addiction in female students is significantly higher than that in male students, which is consistent with the results of previous studies [43]. In some previous studies, there was evidence showing that males were more likely to experience internet addiction than females, while females were more likely to have smartphone addiction than males [63]. (3) There is no gender difference in core self-evaluation, which may be influenced by gender equality advocated in modern society. Women’s self-evaluation is correspondingly improved during the realization of their life value [64].

Moreover, this study found that gender had an insignificant moderating effect. For both male and female college students, physical exercise can not only reduce college students’ smartphone addiction directly, but also reduce smartphone addiction indirectly by enhancing students’ core self-evaluation level. Although male and female students have different levels of physical activity, smartphone addiction, and core self-evaluation, the relationships among these three variables may be so robust that they cannot be moderated by gender. This hypothesis requires further studying and testing.

### 4.4. Significance and Limitations

This study investigated the intrinsic mechanism of physical exercise’s effect on college students’ smartphone addiction by building a mediating model between physical exercise, core self-evaluation, and smartphone addiction, and discovered that physical exercise can influence smartphone addiction through the mediating effect of core self-evaluation. The findings of this study suggest that we should attach great importance to the influence of physical exercise and core self-evaluation on the control and prevention of college students’ smartphone addiction. All colleges and universities should place great importance on reducing students’ smartphone addiction by exploring various measures, including physical exercise. In addition, psychologists and educators in related fields should also pay more attention to the core self-evaluation level of college students and provide professional counseling and guidance for college students in terms of core self-evaluation.

However, due to the limited conditions, there are also some deficiencies in this study that should be addressed in future studies. First, this study mainly examines the mediating effect of core self-evaluation. However, there are more variables influencing the relations between physical exercise and college students’ smartphone addiction. These variables also need further studies, such as the mechanism of meaning in life. Second, the results are derived from Chinese college students, so there are limitations to promote these findings to Western cultures. Therefore, future researchers can study adolescents in different cultural groups. Third, given that the data in this study were entirely self-reported, a variety of other measures should be utilized in the future, even though Harman’s one-factor test indicated that there was no significant common method bias. Finally, this study used a lateral design and failed to deeply reveal the relationships between several variables from a longitudinal perspective. Future studies can try to use cross-lagged regression analysis to explore the relationships among smartphone addiction, physical exercise, and the core self-evaluation of college students, and further test the stability of the current relationship so as to provide a theoretical basis for the effective prevention and intervention of the problem of smartphone addiction in college students.

## 5. Conclusions

This study examined the relationship between physical exercise and smartphone addiction among college students and the mediating effect of core self-evaluation on this relationship. The findings suggest that physical exercise can not only reduce college students’ smartphone addiction directly, but can also reduce it indirectly by enhancing students’ core self-evaluation level. Theoretically, this study is helpful for exploring the internal mechanism of physical exercise on college students’ smartphone addiction and enriches the research results in this field. In practice, this study can help people to take an active initiative to prevent and intervene in smartphone addiction from multiple perspectives and provides empirical research evidence to prevent and reduce smartphone addiction among college students.

## Figures and Tables

**Table 1:** Gender differences for physical exercise, smartphone addiction, and core self-evaluation.

Variable	*M ± SD* (Male)	*M ± SD* (Female)	*t*
1. Physical exercise	26.73 ± 24.57	12.33 ± 15.90	8.53 ***
2. Smartphone addiction	57.37 ± 15.55	59.73 ± 14.32	1.97 *
3. Core self-evaluation	35.48 ± 7.05	35.11 ± 6.00	0.71

* *p* < 0.05, *** *p* < 0.001.

**Table 2:** Descriptive statistics and correlation results for each variable.

Variable	*M ± SD*	1	2	3
1. Physical exercise	20.46 ± 22.39	1		
2. Smartphone addiction	58.40 ± 15.06	−0.30 **	1	
3. Core self-evaluation	35.32 ± 6.61	0.25 **	−0.52 **	1

** *p* < 0.01

**Table 3:** Test for mediating effects.

Effect Type	Path	95% Confidence Interval	Effect Value	Effect Size
Direct effect	Physical exercise → Smartphone addiction	[−0.24, −0.09]	−0.17	58.62%
Mediating effect	Physical exercise → Core self-evaluation → Smartphone addiction	[−0.17, −0.07]	−0.12	41.38%
Total effect		[−0.37, −0.21]	−0.29	100%

## Data Availability

The data presented in this study are available on request from the corresponding author.
